# Diffusional Dynamics of Hydride Ions in the Layered Oxyhydride SrVO_2_H

**DOI:** 10.1021/acs.chemmater.1c00505

**Published:** 2021-04-12

**Authors:** Rasmus Lavén, Ulrich Häussermann, Adrien Perrichon, Mikael S. Andersson, Michael Sannemo Targama, Franz Demmel, Maths Karlsson

**Affiliations:** † Department of Chemistry and Chemical Engineering, Chalmers University of Technology, Göteborg SE-412 96, Sweden; ‡ Department of Materials and Environmental Chemistry, Stockholm University, Stockholm SE-10691, Sweden; § ISIS Facility, Rutherford Appleton Laboratory, Harwell Oxford, Didcot, Oxfordshire OX11 0QX, U.K.

## Abstract

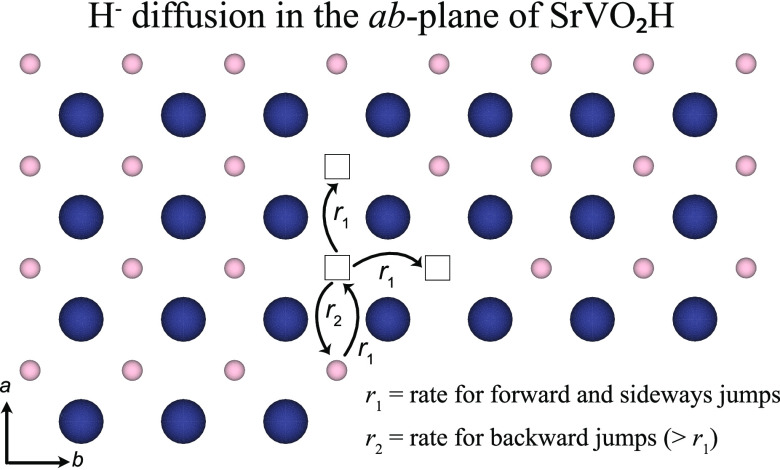

Perovskite-type oxyhydrides are hydride-ion-conducting materials of promise for several types of technological applications; however, the conductivity is often too low for practical use and, on a fundamental level, the mechanism of hydride-ion diffusion remains unclear. Here, we, with the use of neutron scattering techniques, investigate the diffusional dynamics of hydride ions in the layered perovskite-type oxyhydride SrVO_2_H. By monitoring the intensity of the elastically scattered neutrons upon heating the sample from 100 to 430 K, we establish an onset temperature for diffusional hydride-ion dynamics at about 250 K. Above this temperature, the hydride ions are shown to exhibit two-dimensional diffusion restricted to the hydride-ion sublattice of SrVO_2_H and that occurs as a series of jumps of a hydride ion to a neighboring hydride-ion vacancy, with an enhanced rate for backward jumps due to correlation effects. Analysis of the temperature dependence of the neutron scattering data shows that the localized jumps of hydride ions are featured by a mean residence time of the order of 10 ps with an activation energy of 0.1 eV. The long-range diffusion of hydride ions occurs on the timescale of 1 ns and with an activation energy of 0.2 eV. The hydride-ion diffusion coefficient is found to be of the order of 1 × 10^–6^ cm^2^ s^–1^ in the temperature range of 300–430 K, which is similar to other oxyhydrides but higher than for proton-conducting perovskite analogues. Tuning of the hydride-ion vacancy concentration in SrVO_2_H thus represents a promising gateway to improve the ionic conductivity of this already highly hydride-ion-conducting material.

## 1 Introduction

Hydrogen dynamics play a key role in many oxides of high interest to science and society and have been therefore studied from many different points of view. In most cases, hydrogen is present as interstitial protonic (H^+^) defects, which are bonded covalently to lattice oxygens. At elevated temperatures, the O–H bond may break, as a consequence of the thermal energy and intensified vibrational dynamics, to allow the jump diffusion of protons from one oxygen to a neighboring one, leading to long-range proton conductivity.^1^ This dynamical process is well established.^2^ In rare cases, the hydrogen can also be present as substitutional hydride ions (H^–^) on the lattice oxygen sites, thus forming, the so-called, oxyhydrides. Recently, Kobayashi et al.^3^ have demonstrated pure hydride-ion conductivity in La_2–*x*–*y*_Sr_*x*+*y*_LiH_1–*x*+*y*_O_3–*y*_, and La_2_LiHO_3_ has been subsequently investigated in more detail with respect to hydride-ion dynamics and diffusion. Based on first-principles calculations and inelastic neutron scattering data, it was found for La_2_LiHO_3_ that hydride-ion diffusion in the rock salt layer was greatly hindered by the presence of covalent bonding, forcing in-plane hydride-ion diffusion in the perovskite layer to be the dominant transport mechanism.^4^ At variance with the La_2–*x*–*y*_Sr_*x*+*y*_LiH_1–*x*+*y*_O_3–*y*_ system, the majority of oxyhydrides are based on transition metals, such as the solid solutions ATiO_3–*x*_H_*x*_ (A = Ba, Sr, and Ca; *x* ≤ 0.6) that show mixed ionic–electronic conductivity^5,6^ and the stoichiometric compound SrCrO_2_H.^7^ These materials adopt an average cubic perovskite structure with a randomly disordered arrangement of O^2–^ and H^–^ ions, whereas hydrogen-ordered structures have been reported for the layered structured SrVO_2_H^8^ and LaSrCoO_3_H_0.7_^9^ and for the recently discovered 4d transition metal oxyhydrides LaSr_3_NiRuO_4_H_4_ and LaSrCo_0.5_Rh_0.5_O_3_H.^10,11^

Transition metal oxyhydrides have created excitement for their electron transport, mixed ionic–electronic conductivity, and magnetic and catalytic properties.^12^ The hydride ion in transition metal oxyhydrides is considered labile, which allows easy exchange for other ions and also explains the catalytic activity of these compounds. However, in contrast with La_2_LiHO_3_, there is little knowledge on the dynamical properties of hydride ions in transition metal oxyhydrides.

The initial work on dynamical investigations of transition metal oxyhydrides focused on LaSrCoO_3_H_0.7_ by means of quasi-elastic neutron scattering (QENS).^13^ The QENS results revealed the presence of hydride-ion diffusion near the decomposition temperature (*T* ≥ 675 K) that could be described as a vacancy-assisted hydride-ion hopping mechanism along the *a*-axis of the crystal structure. Subsequent studies of hydride-ion dynamics in oxyhydrides have focused primarily on ATiO_3–*x*_H_*x*_ (A = Ba and Sr) using a variety of techniques and with varying results.^14−18^ Analyses of gaseous hydrogen release/exchange experiments and theoretical simulations of BaTiO_3–*x*_H_*x*_ have been interpreted in terms of a hydride-ion diffusion mechanism that is highly dependent on the concentration of hydride ions (*x*) with the simultaneous movement of oxygen ions for *x* < 0.4, whereas for *x* > 0.4, only the hydride ions diffuse, both processes with an activation energy in the range of 1.85–2 eV.^15^ However, in another theoretical study, an activation energy of 0.28 eV was reported for oxygen-vacancy-mediated hydride-ion diffusion.^16^

Most recently, some of us studied the hydride-ion dynamics in metal-hydride-reduced BaTiO_3_ samples characterized by the simultaneous presence of hydride ions and oxygen vacancies, denoted as BaTiO_3–*x*_H_*y*_□_*x*–*y*_ where □ are the oxygen vacancies, using QENS.^17^ Analysis of the QENS data revealed the presence of a temperature-dependent diffusion mechanism characterized by hydride-ion jumps between the nearest-neighbor oxygen vacancies with a mean residence time of the order of 0.1 ns at *T* = 225 and 250 K and with the (exclusive or additional) presence of hydride-ion jumps between the next-nearest-neighboring oxygen vacancies at higher temperatures (*T* > 400 K). A diffusion constant was extracted from the QENS data and takes on values of about 0.4 × 10^–6^ cm^2^ s^–1^ at 225 K and between ca. 20 × 10^–6^ and 100 × 10^–6^ cm^2^ s^–1^ at temperatures between 400 and 700 K. The activation energies were derived from the measurements at high temperatures and take on values of about 0.1 eV and showed a slight increase with increasing oxygen-vacancy concentration.

**Figure 1:**
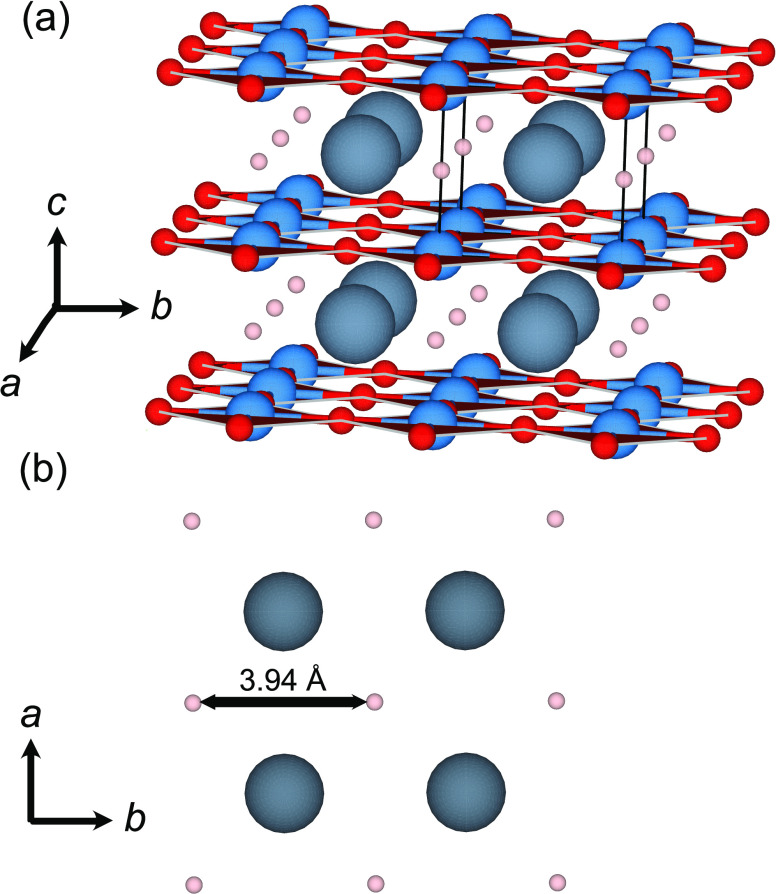
(a) Schematic illustration of the layered crystal structure of SrVO_2_H according to ref (8). Sr, V, O, and H atoms are depicted as gray, blue, red, and pink spheres, respectively. The black lines indicate a unit cell. (b) Schematic illustration of the *ab*-plane in which the hydride ions are coordinated to Sr in a square-planar fashion. The distance between the nearest-neighboring hydride ions in the *ab*-plane is 3.94 Å at ambient temperature and is indicated in the figure. The images were generated using the VESTA software.^19^

In this work, QENS is used to investigate the hydride-ion dynamics in the recently discovered transition metal oxyhydride SrVO_2_H, which is featured by a layered and fully ordered hydride-ion sublattice.^8^ Specifically, SrVO_2_H adopts a tetragonal crystal structure (space group *P*4/*mmm*) and consists of V^3+^ cations coordinated by four O^2–^ in a square-planar fashion. These planes of VO_2_ are connected by planes of Sr–H. The structure is thus described by a VO_2_–SrH −VO_2_–SrH stacking sequence and the fact that the hydride ions lie in the *ab*-plane (see Figure 1) suggests that the hydride-ion diffusion mechanism in SrVO_2_H is different from that in LaSrCoO_3_H_0.7_ and BaTiO_3–*x*_H_*y*_□_*x*–*y*_. The aim of the study thus is to investigate the nature of hydride-ion diffusion in SrVO_2_H and to compare the results with LaSrCoO_3_H_0.7_ and BaTiO_3–*x*_H_*y*_□_*x*–*y*_, as well as to relevant proton-conducting oxides.

## 2 Experimental Details

### 2.1 Sample Synthesis and Characterization

The synthesis of SrVO_2_H powder followed essentially the original report by Denis Romero et al.^8^ About 5 g of SrVO_3_—which was prepared from SrCO_3_ (ABCR, 99.9% purity) and V_2_O_5_ (Sigma-Aldrich, 99.6% trace metal basis) according to the method described by Rey et al.^20^ —was mixed thoroughly with 0.9 g of CaH_2_ (Sigma-Aldrich, 99.9% purity), that is, the molar ratio of H to SrVO_3_ was about 1.6:1, and grinded together for 20 min in an agate mortar. The mixed powder was pressed into pellets with a diameter of 9 mm, which were sealed in a stainless steel autoclave. The whole operation was carried out in an Ar-filled glovebox. The autoclave was then transferred outside the glovebox, pressurized with 20 bar H_2_, and heated in a vertical tube furnace at 600 °C for 2 days. In order to obtain a single-phase tetragonal product, the procedure had to be repeated twice. This involved regrinding the pellets and the addition of extra CaH_2_. As the last step, the sample was sonicated four times with batches of 50 ml of 0.1 M acetic acid to remove CaO and excess CaH_2_ and then washed with pure water and ethanol. The so-purified sample was dried at 120 °C under dynamic vacuum (<10^–5^ bar). The final amount of the product was about 3 g. Powder X-ray diffraction patterns were collected on a Panalytical X’Pert PRO diffractometer operated with Cu Kα radiation and in θ–2θ geometry. The powder sample was mounted in a Si wafer zero-background holder and diffraction patterns were obtained in a 2θ range of 10–120° with 0.016° step size. The Rietveld method as implemented in the FullProf program^21^ was used for phase and structure analyses. A five-coefficient polynomial function was applied for the background and the peak shape was described by a pseudo-Voigt function. The Rietveld analysis confirmed that the observed structure is in good agreement with the one in the original report^8^ (Figure S1).

### 2.2 Quasi-Elastic Neutron Scattering

For the QENS measurements, three different instruments were used, namely, the high-flux backscattering spectrometer (HFBS),^22^ and the disk chopper spectrometer (DCS)^23^ at the NIST Center for Neutron Research, and the time-of-flight near-backscattering spectrometer OSIRIS^24^ at ISIS Neutron and Muon Source. After general and instrument-specific data reduction that is briefly outlined below, the obtained quantity in all these QENS measurements is the measured dynamical structure factor *S*(*q*,*E*), where *q* and *E* are the momentum and energy transfer, respectively. The complementarity in using these three instruments is that they allow us to probe different parts of the (*q*,*E*) space with different resolutions, which translate into a larger range of probed length and timescales, respectively. A 2.8 g powder sample of SrVO_2_H was filled inside a sachet of Al foil under ambient atmosphere. The sachet was put in a hollow cylindrical Al cell, which was used for all measurements.

HFBS was set up using a nominal neutron wavelength of 6.721 Å and with the Doppler drive frequency set to 22 Hz, which together with the Si(111) analyzers yielded an accessible energy transfer range of ±15 μeV and an energy resolution at full width at half-maximum (fwhm) of ≈0.8 μeV at the elastic line. The accessible *q*-range was 0.6–1.6 Å^–1^ at the elastic line. QENS measurements were performed at the temperatures *T* = 330, 370, 400, and 430 K, that is, well below the decomposition temperature at *T* ≈ 550 K, see Figure S2. The resolution function of the instrument was approximated by a measurement of the sample at *T* = 100 K. The standard corrections of the scattering intensity were applied to the data within the DAVE software.^25^

DCS was set up using an incident neutron wavelength of 8 Å, yielding an energy resolution at fwhm of 29.8 μeV at the elastic line and an accessible energy transfer range of ±0.3 meV. The accessible *q*-range was 0.2–1.4 Å^–1^ at the elastic line. QENS measurements were performed at the temperatures *T* = 280, 330, 370, 400, and 430 K. In addition, measurements with incident neutron wavelengths of 5.5 Å (energy resolution fwhm at the elastic line of 38.6 μeV, *q*-range of 0.2–2.1 Å^–1^, and *E* range of ±0.7 meV) and 4.8 Å (energy resolution fwhm at the elastic line of 118.2 μeV, *q*-range of 0.2–2.3 Å^–1^, and *E* range of −5 to 2 meV) were performed at one temperature (*T* = 430 K), in order to probe the dynamical structure factor *S*(*q*,*E*) over a larger range of (*q*,*E*) space. The resolution function of the instrument was approximated by a measurement of the sample at *T* = 4 K. Standard corrections were applied to the time-of-flight data within the DAVE software^25^ and included normalization to a vanadium standard, background subtraction (empty sample cell and dark count measurements), and correction for the energy-dependent efficiency of the detectors.

OSIRIS was set up using the PG(002) analyzers yielding an energy resolution of 25.4 μeV at fwhm and an accessible energy transfer range of ±0.5 meV. The accessible *q*-range was 0.18–1.8 Å^–1^ at the elastic line. QENS measurements were performed at *T* = 100, 150, 200, 250, 300, 350, 380, and 415 K. The resolution function of the instrument was approximated by a measurement of the sample at *T* = 10 K. In addition, neutron diffraction patterns were collected using the OSIRIS diffraction detectors at *T* = 10, 100, 200, and 400 K. Standard data reduction was performed within the Mantid software^26^ and comprised normalization to a vanadium standard to correct for the detector efficiency.

## 3 Results

### 3.1 Long-Range Dynamics

**Figure 2:**
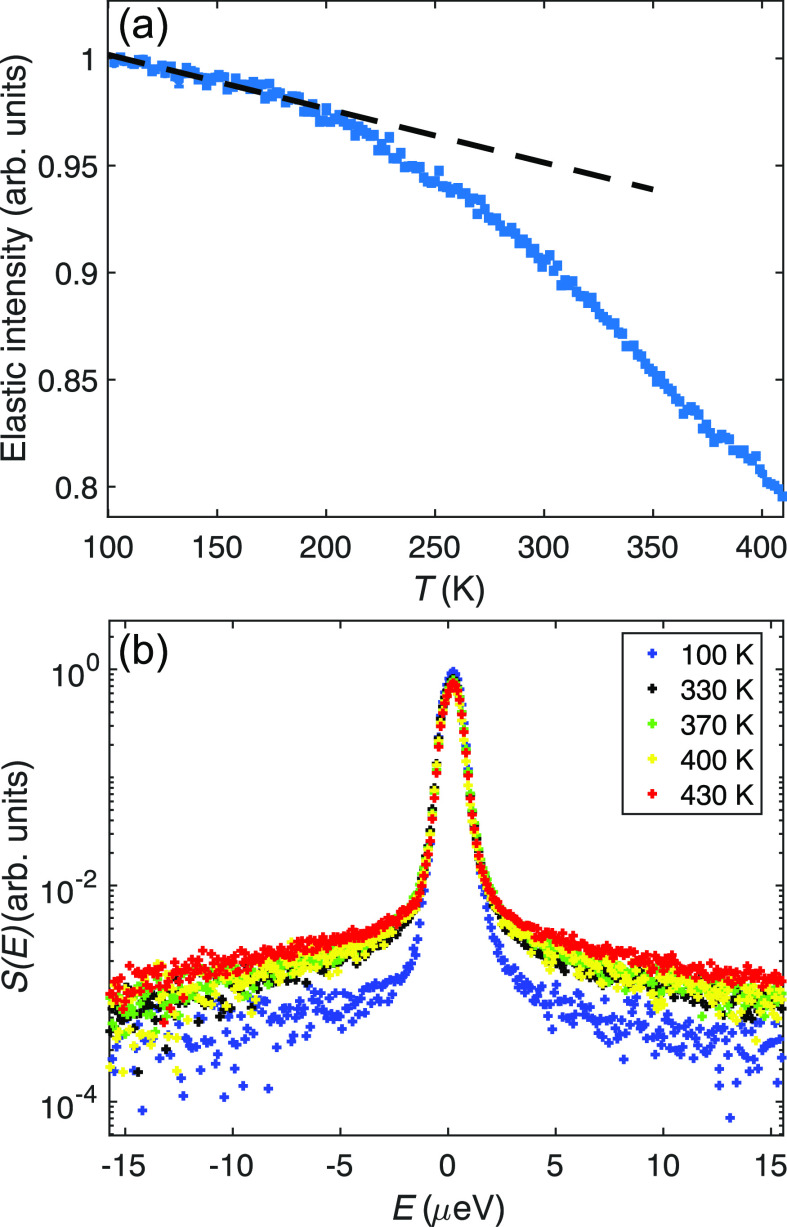
(a) Elastic intensity of SrVO_2_H, as measured on HFBS between 100 and 415 K. The dashed line is a guide to the eye and illustrates the essentially linear behavior for *T* < 200 K. (b) Temperature evolution of the quasi-elastic line shape of SrVO_2_H summed over the *q*-range of 0.6–1.6 Å^–1^.

Long-range dynamics were investigated using the HFBS spectrometer which allows us to probe dynamics on the time range of ≈100 ps to 5 ns. Figure 2a shows the recorded elastic intensity as a function of temperature. For purely harmonic vibrational motions, the elastic intensity is expected to decrease with increasing temperature according to the Debye–Waller factor, with the mean-square displacement increasing linearly with temperature.^27^ Any contributions from relaxational dynamics and/or anharmonic vibrations are expected to give deviations from such a linear behavior. However, since the scattering of SrVO_2_H mainly comes from hydrogen atoms (because of the large incoherent cross section) and the hydrogen vibrations in oxyhydride materials have energies around 100 meV^28^ and therefore only have a small population in the measured temperature range, we do not expect any significant effect from anharmonic vibrations to the data in Figure 2a. A calculation of the Bose–Einstein occupation number *n*(*E*)=[exp(*E*/*k*_B_*T*)–1]^−1^, where *k*_B_ is the Boltzmann constant, for *E* = 100 meV gives *n*(*E*) = 0.07 at the highest measured temperature of *T* = 430 K. At *T* = 200 K, it is only about 3 × 10^–3^, and thus, effects from anharmonic vibrations are indeed negligible in the investigated temperature range. Consequently, the change of slope in the elastic intensity just above *T* = 200 K suggests that some relaxational motion enters the accessible time window of the instrument. The presence of quasi-elastic scattering, observed as a broadening of the elastic peak in *S*(*q*,*E*) for *T* ≥ 330 K, confirms the presence of such motions, see Figure 2b.

**Figure 3:**
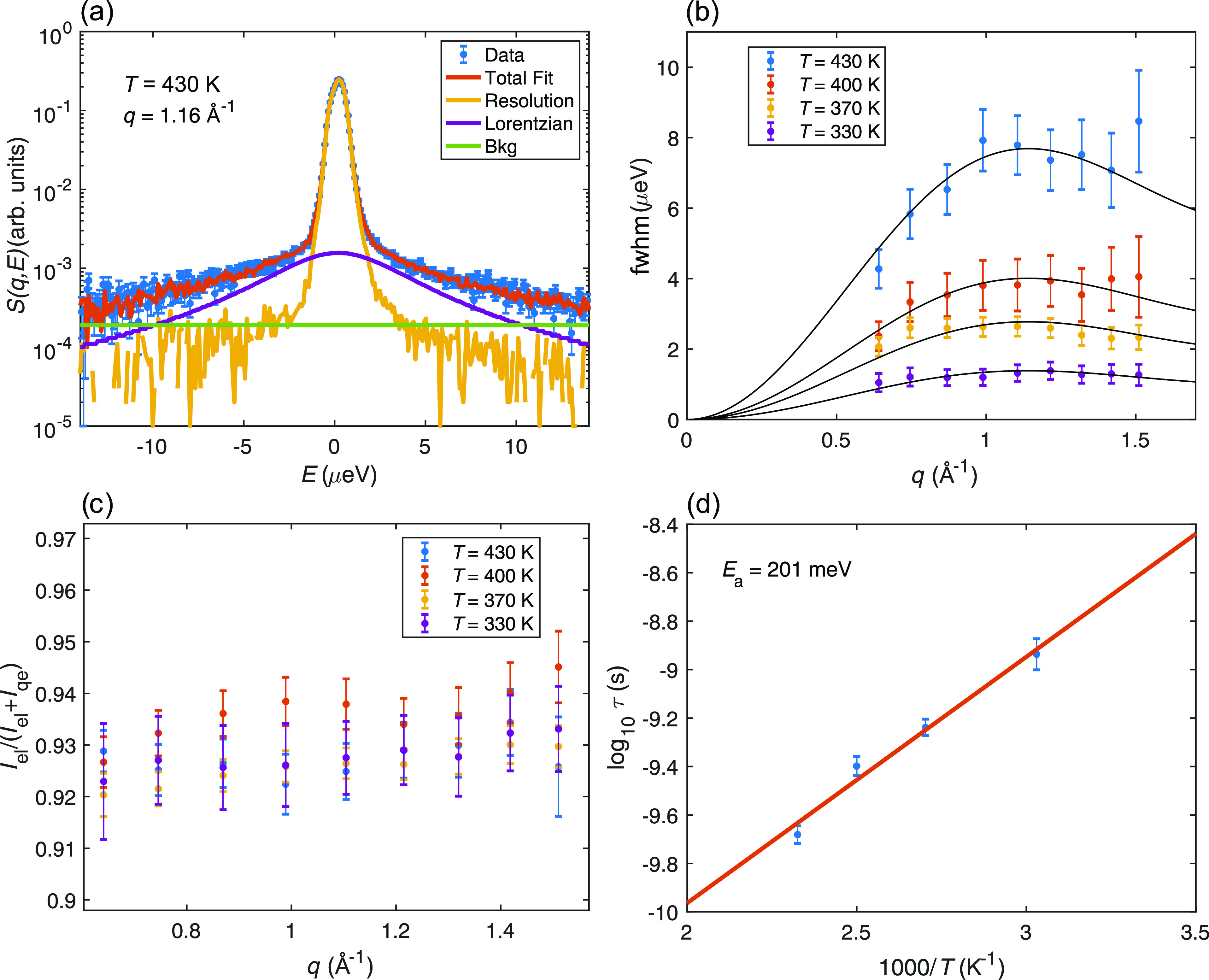
QENS data of SrVO_2_H from neutron backscattering spectroscopy measured on HFBS. (a) Example fitting to the quasi-elastic line shape of SrVO_2_H for *T* = 430 K and *q* = 1.16 Å^–1^. (b) fwhm of the quasi-elastic component as a function of *T* and *q* together with fits to the Chudley–Elliott model describing long-range jump diffusion (solid black lines). (c) Fraction of elastic scattering as a function of *T* and *q*. (d) Fit to an Arrhenius law of the extracted mean residence time τ.

For a quantitative analysis of the relaxational dynamics, *S*(*q*,*E*) was fitted to the following model function
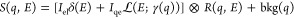
1where *I*_el_ is the elastic scattering intensity, which originates from both hydrogen atoms that move too slowly to be observed on the timescale of the instrument and (incoherent) elastic scattering from the other constituent elements of SrVO_2_H. *I*_qe_ is the quasi-elastic scattering intensity, bkg(*q*) is a flat background that depends on *T* and *q*, *R*(*q*,*E*) is the instrument resolution function, and  is a Lorentzian function with fwhm γ(*q*). Figure 3a shows a fit of *S*(*q*,*E*) for *T* = 430 K and *q* = 1.16 Å^–1^. As can be seen, there is a good fit of the model function to the experimental data, which means that the quasi-elastic scattering can be adequately described by a single Lorentzian function. This was the case for all measured temperatures and *q* values.

Analysis of the fitting results shows that the fwhm of the Lorentzian function increases with *q* [Figure 3b], whereas the fraction of elastic scattering [*I*_el_/(*I*_el_ + *I*_qe_)] is essentially *q*-independent [Figure 3c]. This is in accordance with the established models of a long-range translational jump diffusion process. Crucially, the fwhm can be well approximated with an isotropic average of the well-known Chudley–Elliott jump diffusion model^29^
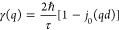
2where *j*_0_ is the zeroth-order spherical Bessel function, τ is the mean residence time between successive jumps, and *d* is the jump distance. It may be noted that, in principle, one should do the powder average for the whole *S*(*q*,*E*). However, based on the previous QENS studies on oxyhydrides,^13,17^ the chosen model in eq 2 can most likely be used as a good approximation and still give reliable estimates of the physical parameters. The jump distance was set to 3.94 Å to reflect the nearest-neighbor H–H distance within the *ab*-planes of SrVO_2_H,^8^ so that only τ was fitted as a free parameter. The data are in good agreement with the model, as can be seen in Figure 3b, which supports the assumption that the hydride-ion diffusion occurs in the *ab*-plane as jumps between well-defined hydride-ion sites. τ varies from 1.2 ns at the lowest measured temperature (*T* = 330 K) to about 0.2 ns at the highest measured temperature (*T* = 430 K).

Figure 3d shows an Arrhenius plot of τ, from which an activation energy of 201 ± 35 meV was extracted. A diffusion coefficient *D* for the hydride ions can be calculated as
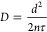
3where *n* is the number of dimensions in which the diffusion occurs.^27^ For SrVO_2_H, *n* = 2 and the calculated diffusion coefficient takes on values between 0.33 × 10^–6^ cm^2^ s^–1^ at *T* = 330 K and 1.85 × 10^–6^ cm^2^ s^–1^ at *T* = 430 K.

With regard to the essentially *q*-independent fraction of elastic scattering, as noted above, it also showed no particular *T*-dependence [Figure 3c]. The fraction of elastic scattering takes on values between 0.91 and 0.94, which means that only about 6–9% of the total scattering intensity is quasi-elastic. Thus, a significant amount of the hydride ions scatter only elastically, that is, their dynamics are too slow to be observed within the dynamical window of the instrument. It should be noted though that also other elements than hydrogen contributes to the elastic scattering of SrVO_2_H. Taking into account the incoherent scattering of all elements in SrVO_2_H, the contribution of elastic scattering that comes from the hydride ions can be recalculated from 0.92 (assumed mean value) to about 0.915. That is, 91.5% of the hydride ions are found to scatter (only) elastically in our experiment.

Last, we note that for diffusion in a plane, which here presumably occurs in the *ab* crystallographic plane, wave vectors (nearly) perpendicular to the diffusion plane give (almost) zero line width.^30^ Thus, this would contribute to an increase of elastic or apparent (smaller line width than the energy resolution of the instrument) elastic scattering. In fact, the powder-averaged *S*(*q*,*E*) has a logarithmic divergence at zero energy transfer for two-dimensional (2D) diffusion. However, when convoluted with the instrument resolution function, this more peaked line shape is hard to distinguish experimentally from a Lorentzian function.^30^

### 3.2 Localized Dynamics

**Figure 4:**
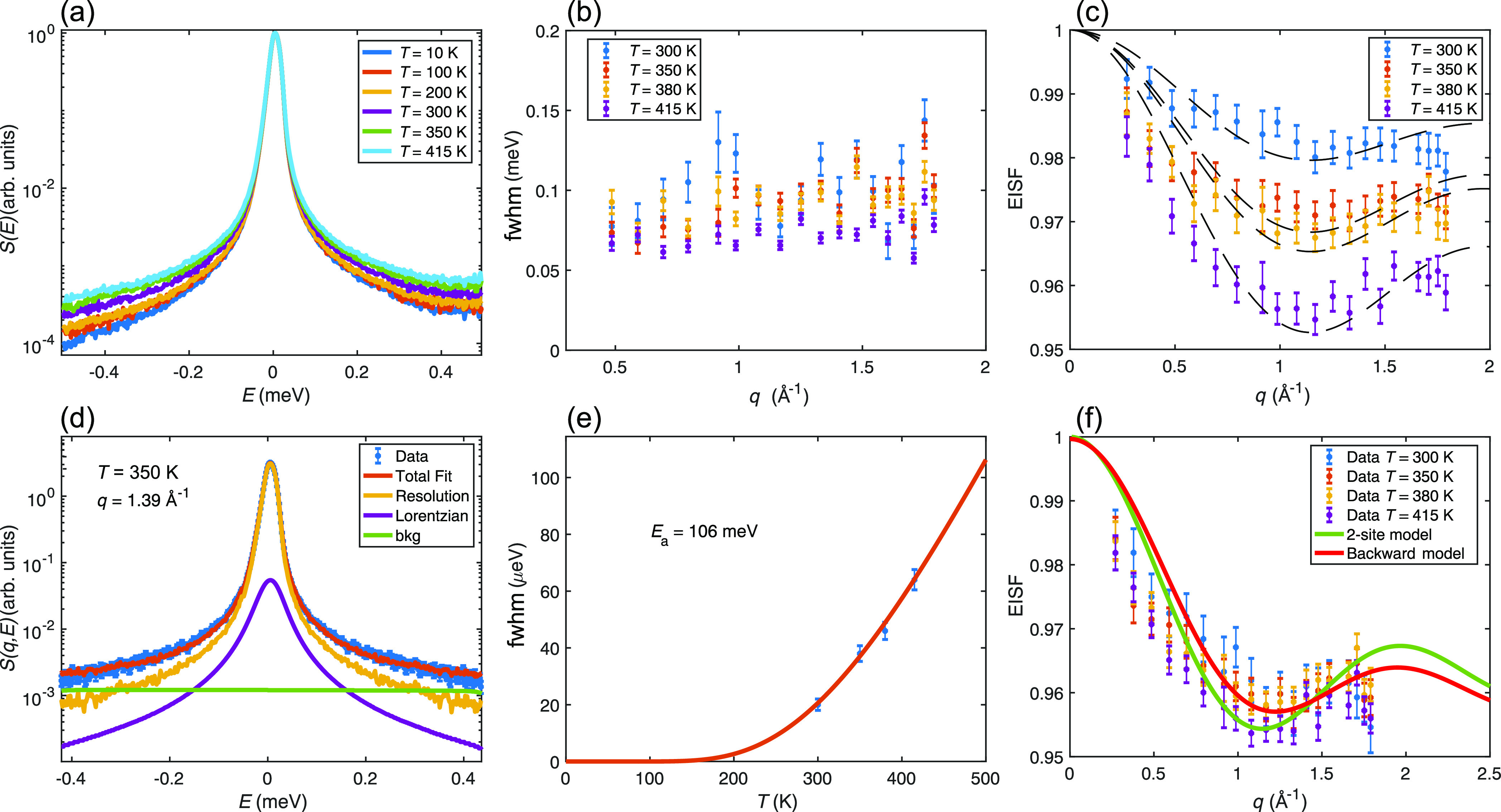
QENS data from OSIRIS. (a) *T* dependence of the quasi-elastic line shape as summed over all *q*-values. (b) Quasi-elastic line width as a function of *q* and *T* as extracted from the “free fits” to the data. (c) Elastic incoherent structure factor (EISF) as a function of *q* and *T* as extracted from the “free fits” to the data. The data were fitted to a two-site jump diffusion model with the jump distance fixed to the H–H distance in the *ab*-plane (3.94 Å). (d) Fit to the quasi-elastic line shape at *T* = 350 K and *q* = 1.39 Å^–1^. (e) Quasi-elastic line width (fwhm) as a function of *T* extracted from the global fits. The solid line is a fit to an Arrhenius law, which yields an activation energy of 106 ± 30 meV. (f) EISF as a function of *q* and *T* extracted from fits with the line width fixed to the value from the “global” fits of *S*(*q*,*E*).

Localized dynamics were investigated on OSIRIS, which allows us to probe dynamics on timescales in the range of ≈1 to 65 ps. Figure 4a shows the temperature dependence of the quasi-elastic line shape as summed over all *q*-values. As can be seen, there is a quasi-elastic broadening that increases with temperature. Fitting of the QENS line shapes revealed that quasi-elastic scattering is present for *T* ≥ 300 K. Below this temperature the line shape could be adequately described by an elastic peak (delta function) and a background, and no obvious QENS signal could be observed (see Figure S5). Like the HFBS data, the *S*(*q*,*E*) spectra from the OSIRIS data can be adequately fitted to an elastic peak, a single Lorentzian function describing the quasi-elastic scattering, and a background, that is, to eq 1. However, for the OSIRIS data, the background needed to be described as a linear function of energy, that is, bkg(*q*,*E*) = α(*q*) + β(*q*)·*E*, where α and β are constants that depend on *q* and *T*.

Our detailed analysis of the QENS data is divided into three steps. In the first step, the data were analyzed by free fits to eq 1. For localized diffusion, the line width (fwhm) γ of the quasi-elastic scattering is expected to be invariant with *q* (or more precisely show a minor *q*-dependence with a non-zero value at *q* = 0), while the relative quasi-elastic scattering intensity is expected to increase with *q*.^27^ Our free fits to the data showed that γ has no particular *q*-dependence [Figure 4b]. Thus, the data indicate that we here probe localized diffusional motions. Compared to the observed dynamics on HFBS, the line width observed on OSIRIS is broader of about a factor of 8–80 depending on temperature. This, together with the different *q*-dependence of the QENS signal, indicates that the here observed QENS signal corresponds to a different dynamical process. Noticeably, the line width showed no particular temperature dependence over the probed temperature region, see Figure 4b. For a classical diffusional process, the line width should increase with increasing temperature according to an Arrhenius relationship. Here, we do not observe this and, in fact, if anything, the line width appears to decrease slightly with increasing temperature. A possible explanation for this is that the line width at lower temperatures is hard to estimate due to the low scattering intensity, while the QENS signal at higher temperatures contains small contributions from the slower dynamical process observed at HFBS, thus leading to an effectively more narrow line width.

For localized diffusion, information on the geometry of the dynamics can be obtained from analyzing the *q*-dependence of the fraction of elastic scattering, which then conventionally is known as the elastic incoherent structure factor (EISF), that is,
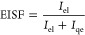


Figure 4c shows the extracted EISF from the free fits to the data for different temperatures. The quasi-elastic scattering intensity increases with temperature and follows a typical *q*-dependence of localized dynamics with a minimum occurring within the probed *q*-range. The minimum occurs around 1.15–1.25 Å^–1^, which would translate into a jump distance between 3.75 and 4.1 Å. Such a jump distance is in full accordance with the nearest-neighbor H–H distance in the *ab*-plane of SrVO_2_H (3.94 Å). The *q*-dependence of the EISF was therefore fitted to a model describing localized jump diffusion between two sites separated by a distance *d*
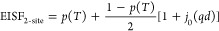
4where *p*(*T*) represents the fraction of immobile (on the timescale of the instrument) hydride ions, and *d* was fixed to the nearest H–H distance in the *ab*-plane (3.94 Å). The EISFs are found to be in good agreement with this model, see Figure 4c. Note that the EISF extracted from the free fits shows a clear temperature dependence. This is in contrast to the HFBS data, for which the quasi-elastic intensity was found to be invariant with temperature. Furthermore, as noted previously, the quasi-elastic line width showed no particular temperature dependence, which is inconsistent with any real physical process. However, as the quasi-elastic scattering signal in this case is only a few percent of the total scattering, the line width and quasi-elastic intensity are strongly correlated variables in the fitting of the QENS spectra. This causes a problem with the “model-free” fitting approach since the “real” line width is hard to estimate reliably due to the small scattering signal. To mitigate this issue, we, in a second step, performed a global fitting procedure of *S*(*q*,*E*) according to a model of localized diffusion. Specifically, *S*(*q*,*E*) was fitted to the following model function
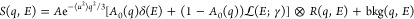
5where *A* is a *q*-independent constant, *e*^–⟨*u*^2^⟩*q*^2^/3^ is the Debye–Waller factor, ⟨*u*^2^⟩ is the isotropic mean-square displacement, and *A*_0_(*q*) is the EISF which was fixed to the expression for a two-site model in eq 4 with *d* = 3.94 Å and *p* = 0.925. The value of *p* was obtained from the fit to the EISF obtained from the “free fit” procedure at 415 K. Note that this value of *p* is also in line with the HFBS data, which showed that only about 7–8% of the hydride ions are mobile. We stress that *S*(*q*,*E*) was fitted globally using eq 5 with only γ, *A*, and ⟨*u*^2^⟩ as *q*-independent parameters and a sloping background. A representative fit to the QENS spectra at 350 K is shown in Figure 4d. The resulting quasi-elastic line width (fwhm) is shown as a function of temperature in Figure 4e. The fwhm follows an Arrhenius behavior with an activation energy of 106 ± 30 meV. As a consistency check, we, in a third step, performed a fitting procedure with the line width fixed to the value obtained in Figure 4e, from which the EISF was extracted. The so-obtained EISFs are shown in Figure 4f for different temperatures. Crucially, the EISFs are temperature independent and in good agreement with the model for localized diffusion between two hydride-ion sites in the *ab*-plane. This suggests that our analysis is robust and that the model function in eq 5 is physically sound.

**Figure 5:**
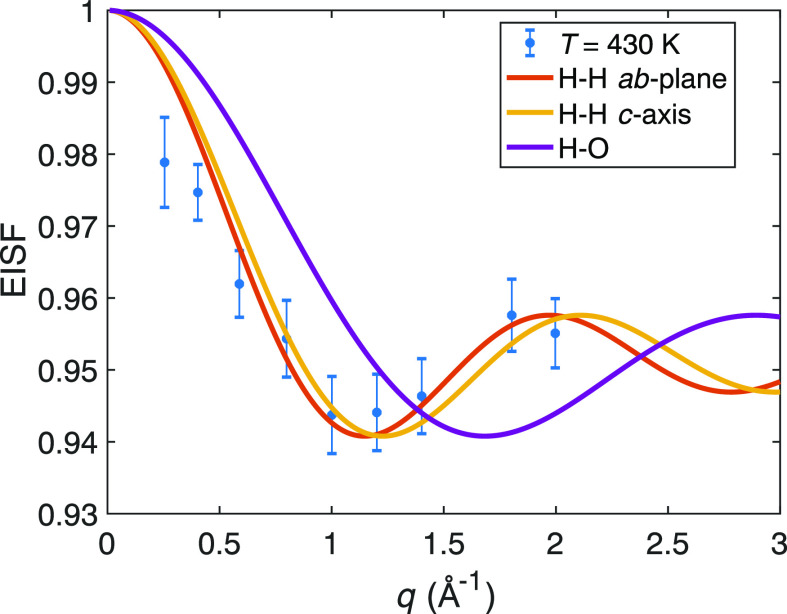
EISF of SrVO_2_H as measured on DCS at 430 K using an incident neutron wavelength of 5.5 Å. The data point at *q* ≈ 1.7 Å^–1^ is removed due to the presence of a nuclear Bragg peak.

In order to discriminate with more accuracy between other possible jump diffusion models describing different plausible diffusion paths in the lattice, QENS spectra at even larger *q*-values were recorded on DCS. Figure 5 shows the EISF at 430 K probed with 5.5 Å wavelength neutrons. The data are compared to jump diffusion models between two sites with three different possible jump distances corresponding to the distance between two neighboring hydride ions in the *ab*-plane (labeled H–H *ab*-plane), the distance between two neighboring hydride ions along the *c*-axis (labeled H–H *c*-axis), and the nearest distance between a hydride and oxygen ion (labeled H–O). As can be seen, the data directly rules out jump diffusion between hydride and oxygen sites. Since the jump distances between the nearest hydride-ion sites along the *c*-axis and in the *ab*-plane are similar (3.66 and 3.94 Å, respectively), both of these models show good agreement with the data, with a slight preference for jumps in the *ab*-plane. Thus, jumps along the *c*-axis can in principle not be ruled out based on the data presented here. However, the hydride ions are along the *c*-axis separated by V atoms between them. Consequently, for jump diffusion to occur along the *c*-axis, the hydride ions need to move past the V atoms, which is likely to require substantially more energy than to move in the *ab*-plane where there is free space between the neighboring hydride-ion sites. Thus, jumps along the *c*-axis can therefore be considered much less likely to occur. Hence, we assign the observed dynamics to jump diffusion of hydride ions between sites in the *ab*-plane.

## 4 Discussion

The observation of two different relaxational processes, that is, slow long-range diffusion of hydride ions between sites in the *ab*-plane and faster localized diffusion between two hydride-ion sites (one of them being vacant), raises the question how these two processes are linked to each other. Of relevance here, we note that SrVO_2_H is believed to be stoichiometric in H,^8^ and, therefore, the concentration of hydride-ion vacancies available for diffusion can be expected to be low. In effect, after one hydride ion has made a jump to a vacant site, the probability of a return jump is likely to be considerably increased because there is a higher (than average) probability to find a vacancy at the original position. This would decrease the residence time for backward jumps, which then would be observable as a localized motion in the experiment.^31^ Hence, such a correlated jump diffusion mechanism—usually neglected in QENS studies of ionic conductors generally—is consistent with our experimental results showing a slow long-range diffusion together with a faster localized motion in SrVO_2_H.

**Figure 6:**
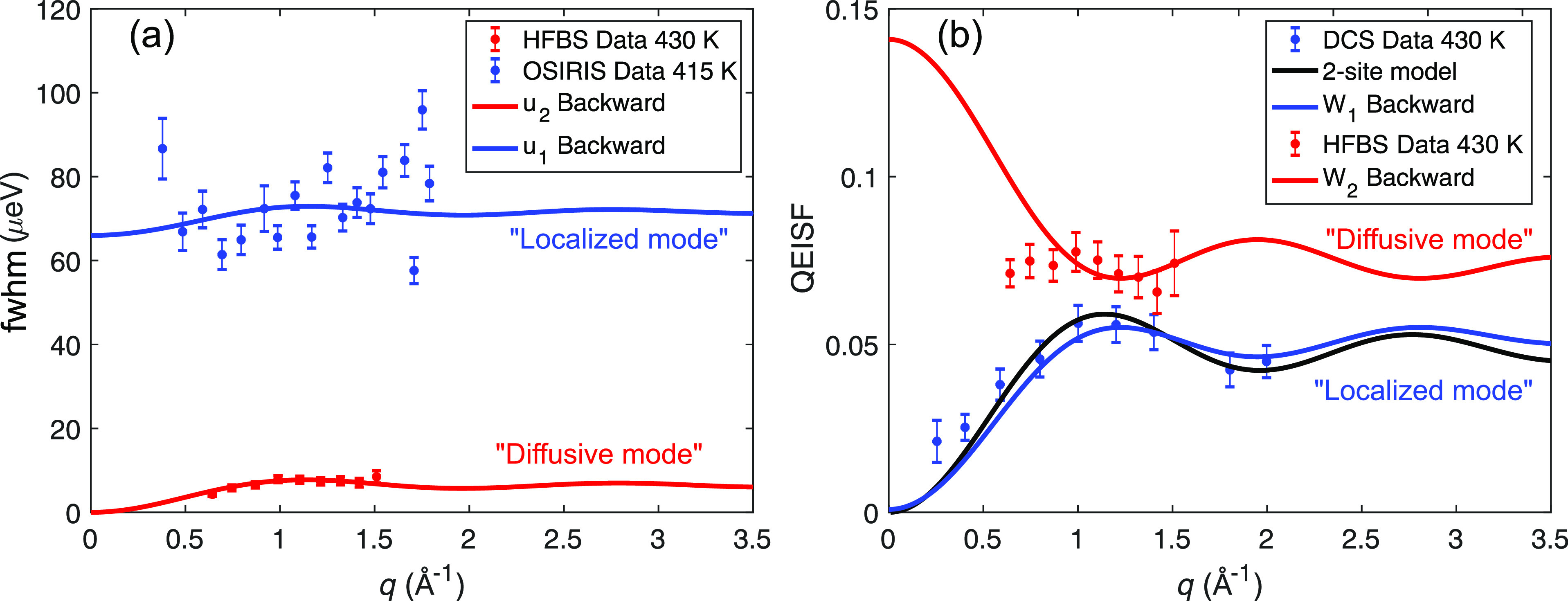
Experimentally determined quasi-elastic line width (fwhm) (a) and quasi-elastic incoherent structure factor (QEISF) (b) as a function of *q* compared to what is predicted for the isotropic average of the backward jump model. *u*_*i*_ and *W*_*i*_, *i* = 1, 2, are the line widths (fwhm) and weights of the two Lorentzian functions in the backward jump model, respectively. The best fit to the data was obtained with 1/τ_1_ ≈ 10/τ_2_. The quasi-elastic structure factors were scaled by a factor 1 – *p*, where *p* represents the immobile (on the timescale of the instrument) fraction of H atoms.

For a quantitative analysis of correlated hydride-ion diffusion within the *ab*-plane of SrVO_2_H, we compare our QENS data to the isotropically averaged backward jump model^32,33^ on a 2D square lattice (Figure 6). This model considers diffusion on a lattice where the jump rate for a backward jump, 1/τ_1_, is larger than the jump rate to any other site, 1/τ_2_, and is explained in mathematical detail in Section S4. The model thus contains some “memory effects”, that is, the diffusion step depends on the previously visited site. The result is that *S*(*q*,*E*) can be, for the 2D square lattice with only nearest-neighbor jumps, described by a sum of two Lorentzian functions, where the dynamics associated with one of these Lorentzian functions are diffusive, that is, the line width goes as *q*^2^ for small *q*, whereas the other Lorentzian function shows a localized characteristic with a larger line width corresponding to the backward jump rate.

Figure 6a shows the *q*-dependence of the line width for the long-range (diffusive) and localized motions, as extracted from the HFBS and OSIRIS experiments, respectively, together with the theoretically predicted *q*-dependence from the powder-averaged backward jump model. As can be seen, the diffusive mode is in excellent agreement with the diffusive mode predicted theoretically, whereas the localized motion is in good agreement with the localized mode in the backward jump model with 10 τ_1_ = τ_2_. These results not only provide support for the correlated jump diffusion model but also show that the probability of a backward jump is a factor of 10 larger than a forward or sideway jump. Considering that in SrVO_2_H the hydride-ion vacancy concentration is most likely low, this seems intuitively reasonable because the probability of finding a vacancy at the previously visited position must be considerably larger than the probability of finding a vacancy at another neighboring site. We speculatively estimate the number of vacancies to be between the fraction of mobile hydride ions and the fraction of mobile hydride ions divided by the number of nearest neighbors (4), which gives a value in the range of 2–8%. One may note that diffraction techniques are not sensitive to such a low concentration of hydride-ion vacancies, which supports that our estimation is reasonable.

For an even more detailed analysis, we compare the QEISFs (QEISF = 1 – EISF) for the diffusive and localized modes to the respective QEISFs as predicted from the backward jump model, see Figure 6b. Here, the localized mode, as described by the backward jump model, is almost identical to the two-site model and is thus in good agreement with the DCS data. For the diffusive mode, we find that the model accounts for the main features of the experimental data, although some slight inconsistency for the lowest *q*-values is observed. Still, even though it seems that the backward model cannot describe our experimental data perfectly, it does explain the main features.

The activation energy for long-range (translational) hydride-ion diffusion was determined to be about 0.2 eV in SrVO_2_H. This value is similar to what was observed in the previous QENS study on the oxyhydride LaSrCoO_3_H_0.7_ by Bridges et al.,^13^ although slightly higher than for BaTiO_3–*x*_H_*x*_ as found by Eklöf-Österberg et al.^17^ The calculated (self) diffusion coefficient takes on values between 0.33 × 10^–6^ cm^2^ s^–1^ at 330 K and 1.85 × 10^–6^ cm^2^ s^–1^ at the highest measured temperature of 430 K. This is roughly 1 order of magnitude lower than what was measured for BaTiO_3–*x*_H_*x*_ at 400 K. It is also more than 1 order of magnitude lower than what was found for LaSrCoO_3_H_0.7_ at above 685 K, that is, above the onset temperature for hydrogen loss, whereas below 685 K, no dynamics were observed.^13^ Of relevance here, it should be noted that Bridges et al.^13^ calculated the diffusion coefficient for three-dimensional diffusion while still claiming the hydride-ion diffusion in LaSrCoO_3_H_0.7_ to be uniaxial. Their values thus reflect the average diffusion coefficient over all crystal directions, while the hydride-ion self-diffusion coefficient for uniaxial diffusion is larger by a factor of 3, thus making it almost 2 orders of magnitude larger than what we here observe for SrVO_2_H. Extrapolating our results for the diffusion coefficient of SrVO_2_H from the Arrhenius fit to 685 K, however, gives a diffusion coefficient of about 1.4 × 10^–5^ cm^2^ s^–1^, which is still a factor of 4 less than what was observed for LaSrCoO_3_H_0.7_ at 685 K. This points toward a complex relationship between the nature of hydride-ion diffusion mechanisms in oxyhydrides of different types.

At this point, we also compare our results to proton-conducting perovskite analogues. Typical values of the diffusion coefficient for long-range proton diffusivity in proton-conducting perovskites extracted from QENS experiments are of the order of 1 × 10^–6^ cm^2^ s^–1^ for temperatures around 700 K.^34−36^ This is similar to what we have found for SrVO_2_H at the lower temperature range of 300–430 K as studied here. In effect, this suggests that SrVO_2_H may be exploited in low-/intermediate-temperature (≈200–500 K) technological applications based on ion-conducting systems that are not possible with the use of proton-conducting materials because of their generally higher working temperatures.

Last, one should note that the above values of the hydride-ion diffusivity do not a priori reflect neither the concentration of mobile species nor the concentration of the nearby vacant sites in the respective material. Nevertheless, the hydride-ion diffusivity is likely to be a function of both the hydride-ion concentration and the concentration and location of hydride-ion and oxide-ion vacancies in the material, which raises the question whether there is any optimum ratio of hydride ions and hydride-ion and oxide-ion vacancies for achieving the highest possible hydride-ion diffusivity and/or macroscopic conductivity. Further research in this direction is likely to be rewarding.

## 5 Conclusions

The hydride-ion dynamics in the oxyhydride SrVO_2_H have been studied by QENS. Our results show that the hydride ions undergo 2D vacancy-assisted jump diffusion restricted to the hydride-ion sublattice of SrVO_2_H. Specifically, the long-range translational diffusion of hydride ions occurs as a series of jumps of a hydride ion to a neighboring hydride-ion vacancy, with an enhanced rate for backward jumps due to correlation effects. This suggests that tuning of the hydride-ion vacancy concentration in SrVO_2_H may be an effective route toward higher ionic conductivity in this promising material. Quantitatively, the localized jumps of hydride ions are featured by a mean residence time of the order of 10 ps with an activation energy of 0.1 eV, whereas the long-range diffusion of hydride ions occurs on the timescale of 1 ns and with an activation energy of 0.2 eV, and the hydride-ion diffusion coefficient is found to be of the order of 1 × 10^–6^ cm^2^ s^–1^ in the temperature range of 300–430 K. In comparison, this diffusion coefficient is comparable to other oxyhydrides but higher than for relevant proton-conducting oxides.

## Supporting Information Available

X-ray and neutron diffraction data; TG data; fits of QENS spectra; and mathematical details of the backward jump diffusion model (PDF)
