# Supplementary material for: Diffusional Dynamics of Hydride Ions in the Layered Oxyhydride SrVO2H
Source: Chem Mater. 2021 Apr 12;33(8):2967–75. doi: 10.1021/acs.chemmater.1c00505 (PMC8154327; doi:10.1021/acs.chemmater.1c00505)
Supplement: Supplementary file 1 — cm1c00505_si_001.pdf [file cm1c00505_si_001.pdf]

Diffusional Dynamics of Hydride Ions in the Layered Oxyhydride $SrVO_2H$ (Supporting Information)

Rasmus Lavén,[†] Ulrich Häussermann,[‡] Adrien Perrichon,[§] Mikael S. Andersson,[†] Michael Sannemo Targama,[‡] Franz Demmel,[§] and Maths Karlsson[*†]

[†]Department of Chemistry and Chemical Engineering, Chalmers University of Technology, SE-412 96 Göteborg, Sweden. [*]E-mail: maths.karlsson@chalmers.se. [‡]Department of Materials and Environmental Chemistry, Stockholm University, Stockholm SE-10691, Sweden. [§]ISIS Facility, Rutherford Appleton Laboratory, Harwell Oxford, Didcot, Oxfordshire OX11 0QX, United Kingdom.

S1 Sample Characterization

Fig. S1 shows the Rietveld fit to the PXRD data of $SrVO_2H$. The observed structure is in good agreement with the one in the original report,[S1] *i.e.* a layered tetragonal structure ($P4/mmm$), with lattice parameters a = 3.935 Å, and c = 3.666 Å at ambient temperature. The refinement details are reported in Table S1. Fig. S2 shows thermal gravimetric (TG) data of $SrVO_2H$ as measured under ambient atmosphere. It can be seen that the sample is stable up to about 570 K after which oxidation occurs.

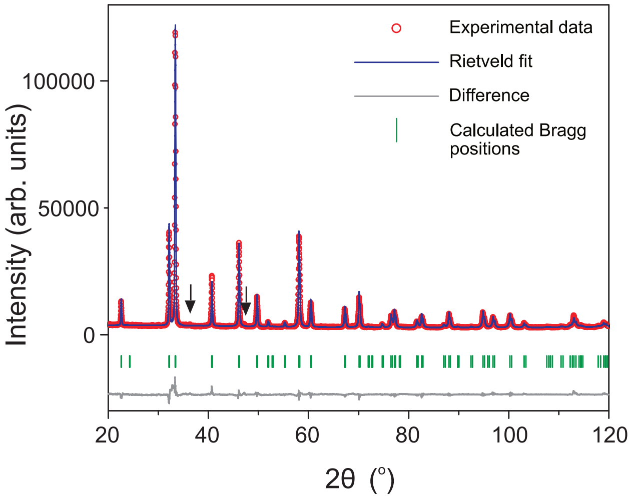

Fig. S1 Rietveld fit to the PXRD data (Cu K_α) of the $SrVO_2H$ sample. The arrows mark intensity from an unknown impurity.

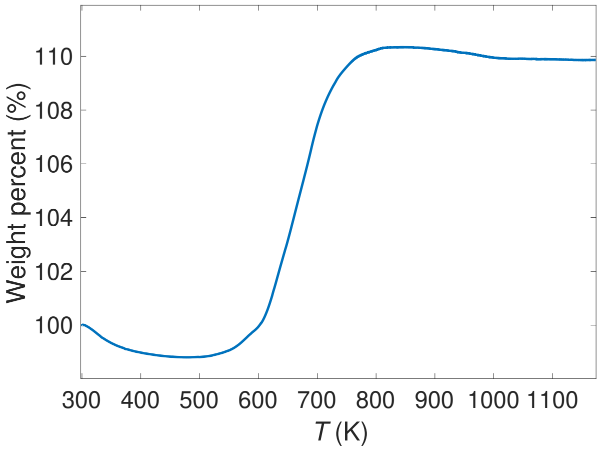

Fig. S2 TG data of $SrVO_2H$, as measured under ambient atmosphere.

S2 Neutron Diffraction

Fig. S3 (a) shows variable temperature neutron powder diffractograms of $SrVO_2H$ as measured on OSIRIS. The diffractograms are in good agreement with the one reported by Romero *et al.*,[S1] confirming the reported layered tetragonal structure for all measured temperatures in the QENS experiment. The blue, green, and red ticks mark nuclear Bragg peaks from $SrVO_2H$, magnetic Bragg peaks from the antiferromagnetic structure of $SrVO_2H$,[S1] and Bragg peaks from the Al sample can, respectively. The observation of the magnetic Bragg peak at ≈ 4.5 Å reveals that the antiferromagnetic ordering persists up to, at least, 400 K. The impurity peaks observed in the PXRD data (Fig. S1) are in the neutron diffraction data manifested as minor peaks (barely visible above the background) at ≈ 2.47 Å and ≈ 1.93 Å. Fig. S3 (b) shows the measured lattice parameters (a and c) as a function of temperature, extracted from the positions of the (100) and (001) nuclear Bragg peaks. Both lattice parameters increase with increasing temperature, with a more rapid rate for c. It follows that the sample becomes slightly more "cubic-like" with increasing temperature.

S3 Fitting of the QENS Lineshape

Fig. S4 and S5 show example fits to the QENS lineshape measured on HFBS and OSIRIS at various temperatures, respectively.

S4 Backward Correlations

Theoretically, correlation effects in relation to solid state diffusion mechanisms have been the subject of numerous studies in

Table S1 Compilation of the data extracted from the Rietveld analysis of the PXRD data of $SrVO_2H$.

Sample	Lattice parameters (Å)	Unit-cell volume (Å^3)	χ^2	R_{Bragg}	R_F	R_{wp}
$SrVO_2H$	a = 3.9352(2), c = 3.6662(3)	56.775(1)	8.95	5.84	4.82	13.7

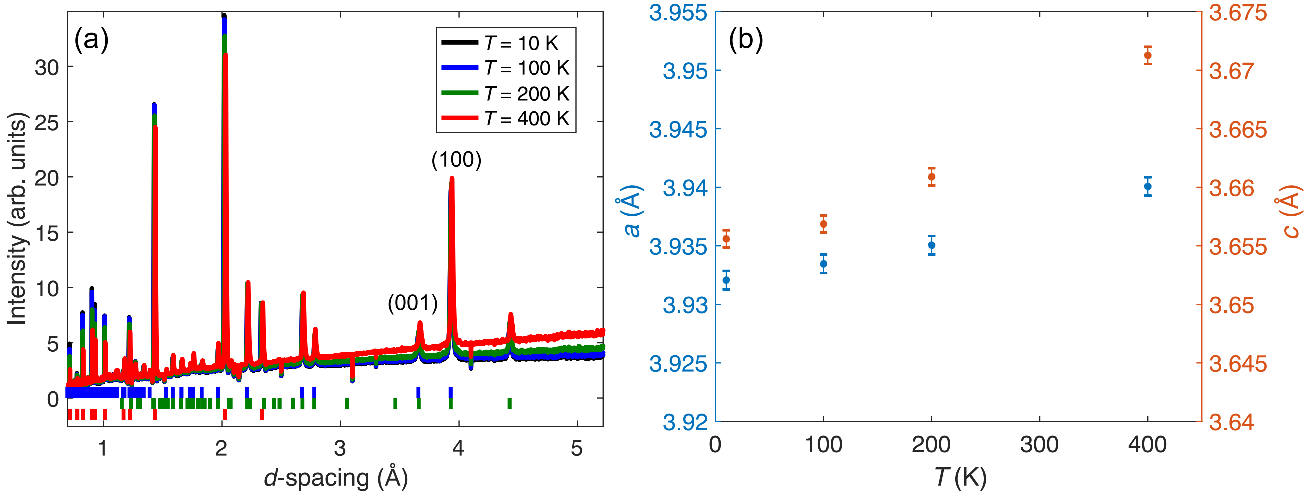

Fig. S3 (a) Neutron powder diffraction patterns obtained at OSIRIS for different temperatures. Blue ticks mark nuclear Bragg peaks from $SrVO_2H$, green ticks correspond to the antiferromagnetic structure of $SrVO_2H$,[S1] and red ticks mark Bragg peaks from the Al sample can. (b) Variable temperature lattice parameters of $SrVO_2H$ as extracted from the OSIRIS diffraction data.

the past,[S2–S8] and it has been noted that correlation effects are generally enhanced for low-dimensional systems,[S8] such as the layered material $SrVO_2H$ that is of concern here.

As a simple phenomenological model to evaluate the QENS data of, and possible correlated jump-diffusion dynamics in, $SrVO_2H$, we choose the so-called backward jump model,[S9] which features an enhanced probability for a backward (return) jump. This model was developed by Haus and Kehr[S9] and the general principle is to separate the self-correlation function $G_s(\mathbf{r},t)$ into components which correspond to the history of the particle:

$$G_s(\mathbf{r},t) = \sum_{i=1}^{z} P(\mathbf{r},\mathbf{r}_i,t), \tag{1}$$

where $P(\mathbf{r},\mathbf{r}_i,t)$ is the probability that the particle is at site $\mathbf{r}$ at time t after previously being at site $\mathbf{r}_i$ one step before and at $\mathbf{r}_0$ at $t=0$. Considering only nearest neighbour jumps, z is the number of nearest neighbour sites. The equations for determining $P(\mathbf{r},\mathbf{r}_i,t)$ may be expressed as[S9]

$$\frac{\partial}{\partial t}P(\mathbf{r},\mathbf{r}_i,t) = -\left(\frac{1}{\tau_1} + (z-1)\frac{1}{\tau_2}\right)P(\mathbf{r},\mathbf{r}_i,t) + \frac{1}{\tau_1}P(\mathbf{r}_i,\mathbf{r},t) + \frac{1}{\tau_2}\sum_{m=1}^{z} P(\mathbf{r}_i,\mathbf{r}_m,t), \tag{2}$$

where $1/\tau_1$ is the return jump rate and $1/\tau_2$ is the jump rate to a new site. After a Fourier transform in space and a Laplace transform in time, Eq. (2) may be expressed in matrix form as[S9]

$$(u - \mathbf{D}(\mathbf{q}))\mathbf{P}(\mathbf{q},u) = 1, \tag{3}$$

where u is the Laplace time and $\mathbf{D}(\mathbf{q})$ is the so-called dynamical matrix and $\mathbf{P}(\mathbf{q},u)$ is the vector with $P(\mathbf{q},\mathbf{r}_i,u), i = 1,2,\ldots,z$ as components. This equation is then readily solved by ordinary eigenvalue methods:

$$P(\mathbf{q},u) = \sum_{n=1}^{z} \frac{W_n(\mathbf{q})}{u - u_n(\mathbf{q})/2}, \tag{4}$$

where $u_n(\mathbf{q})/2$ are the eigenvalues of $\mathbf{D}(\mathbf{q})$, and the weights $W_n(\mathbf{q})$ are given by $W_n(\mathbf{q}) = \sum_{l,m} T_{ln}(T^{-1})_{nm}$, where T is the matrix constructed by the eigenvectors of $\mathbf{D}(\mathbf{q})$ as columns. The dynamical structure factor $S(\mathbf{q},E)$ is related to $P(\mathbf{q},u)$ by the following relation

$$S(\mathbf{q},E) = \frac{1}{\pi}\mathrm{Re}\left[P(\mathbf{q},u=\mathrm{i}E)\right] = \frac{1}{\pi}\sum_{n=1}^{z}\frac{u_n(\mathbf{q})}{2}\frac{W_n(\mathbf{q})}{E^2 + (u_n(\mathbf{q})/2)^2}. \tag{5}$$

$S(\mathbf{q},E)$ is thus a sum of Lorentzian functions with FWHMs $u_n(\mathbf{q})$ and weights $W_n(\mathbf{q})$.

For $SrVO_2H$, we calculated numerically the eigenvalues and eigenvectors of the backward jump model for diffusion on the 2D square lattice. The results are shown in Fig. S6, for the example case when $\tau_2 = 8\,\tau_1$. As can be seen in the figure, there are two non-vanishing modes, one low-energy diffusive mode and one higher-energy localized mode that emerges due to the increased probability of a backward jump.

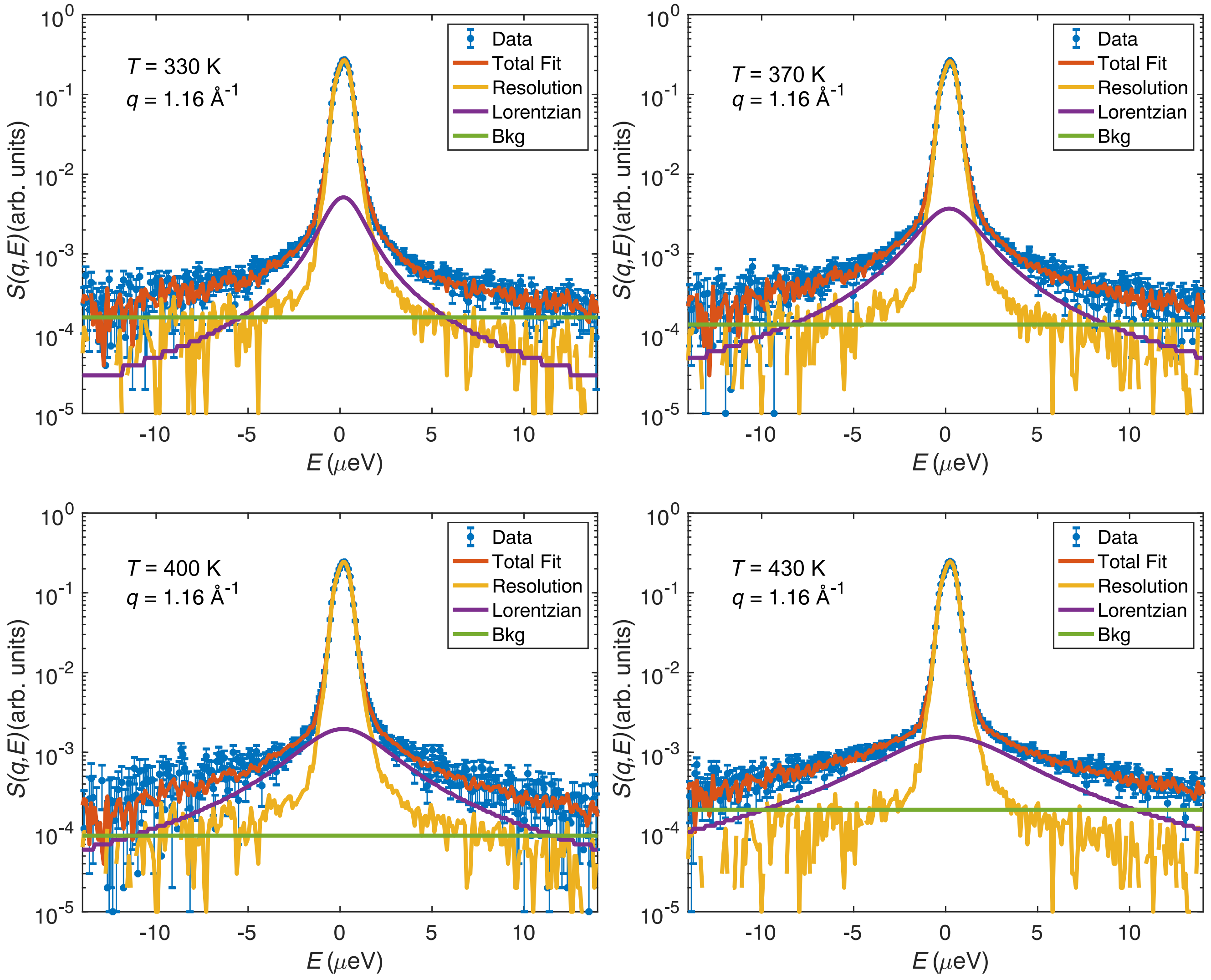

Fig. S4 Example fits to the QENS lineshape measured on HFBS at various temperatures.

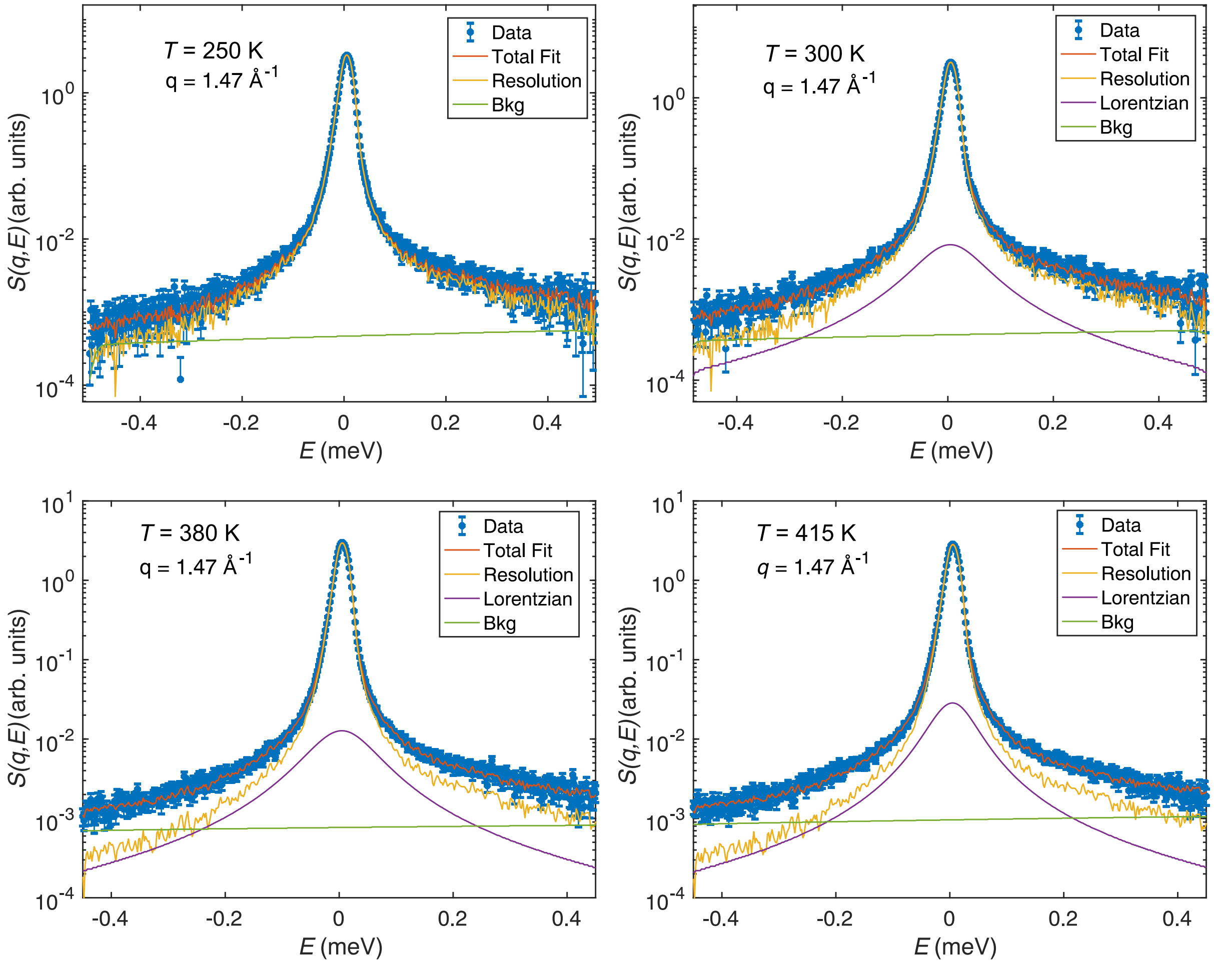

Fig. S5 Example of the free fits to the QENS lineshape measured on OSIRIS at various temperatures. Notice that for $T = 250$ K no QENS could be detected within the experimental errors of the data.

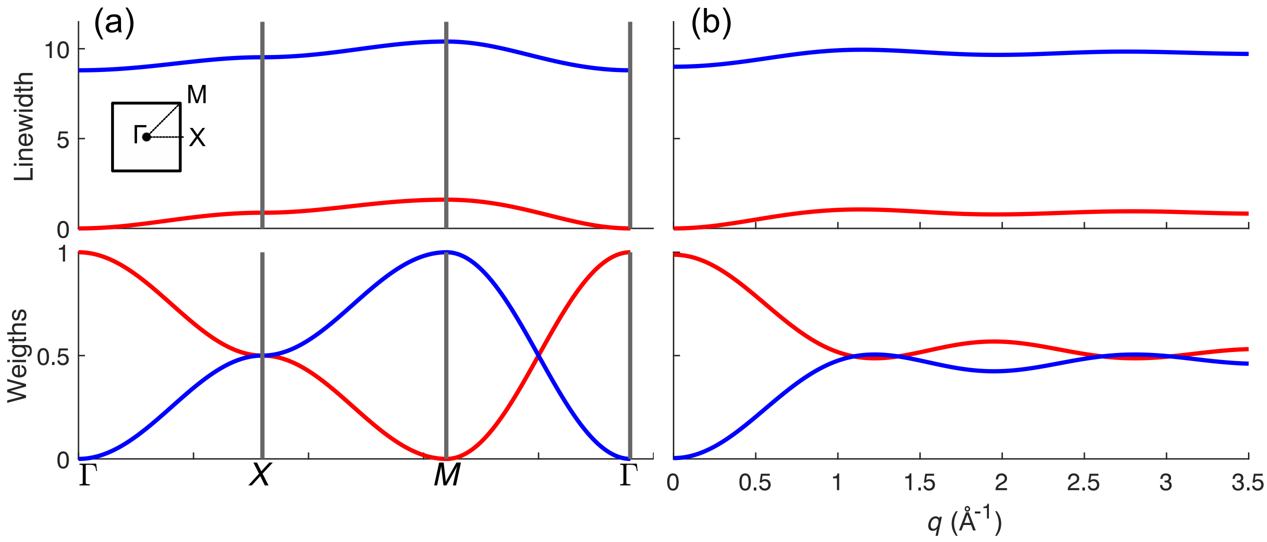

Fig. S6 (a) Dispersion of the eigenvalues and weights of a simple backward jump model along the high-symmetry directions of the 2D square lattice as indicated in the inset. The values were calculated for the example case when $1/\tau_1 = 4$ and $\tau_2 = 8\,\tau_1$. (b) 3D powder average of the corresponding dispersions in (a).

References

(S1) Denis Romero, F.; Leach, A.; Möller, J. S.; Foronda, F.; Blundell, S. J.; Hayward, M. A. Strontium Vanadium Oxide-Hydrides: "Square-Planar" Two-Electron Phases. *Angew. Chemie - Int. Ed.* **2014**, *53*, 7556–7559.

(S2) Kehr, K. W. Diffusion in Concentrated Lattice Gases. *J. Stat. Phys.* **1983**, *30*, 509–518.

(S3) Klamut, J.; Gubiec, T.; Gubiec, T.; Kutner, R. Correlated Hopping in Honeycomb Lattice: Tracer Diffusion Coefficient at Arbitrary Lattice Gas Concentration. *J. Phys. C: Solid State Phys.* **1985**, *18*, 6323–6339.

(S4) Kutner, R.; Binder, K.; Kehr, K. W. Diffusion in Concentrated Lattice Gases. II. Particles with Attractive Nearest-Neighbor Interaction on Three-Dimensional Lattices. *Phys. Rev. B* **1982**, *26*, 2967–2980.

(S5) Kutner, R.; Kehr, K. W. Diffusion in Concentrated Lattice Gases: Intermediate Incoherent Dynamical Scattering Function for Tagged Particles on a Square Lattice. *Phys. Rev. B* **1990**, *41*, 2784–2793.

(S6) Tahir-Kheli, R. A.; Elliott, R. J. Correlated Random Walk in Lattices: Tracer Diffusion at General Concentration. *Phys. Rev. B* **1983**, *27*, 844–857.

(S7) Haus, J. W.; Kehr, K. W. Diffusion in Regular and Disordered Lattices. *Phys. Rep.* **1987**, *150*, 263–406.

(S8) Kehr, K. W.; Kutner, R.; Binder, K. Diffusion in Concentrated Lattice Gases. Self-Diffusion of Noninteracting Particles in Three-Dimensional Lattices. *Phys Rev. B* **1980**, *23*, 4931–4945.

(S9) Haus, J. W.; Kehr, K. W. Random Walk Model With Correlated Jumps: Self-Correlation Function and Frequency-Dependent Diffusion Coefficient. *J. Phys. Chem. Solids* **1979**, *40*, 1019–1025.
